# A multi-modal panel dataset to understand the psychological impact of the pandemic

**DOI:** 10.1038/s41597-023-02438-y

**Published:** 2023-08-11

**Authors:** Isabelle van der Vegt, Bennett Kleinberg

**Affiliations:** 1 Utrecht University, Department of Sociology, Utrecht University, 3584 CH Utrecht, The Netherlands; 2 Tilburg University, Department of Methodology and Statistics, 5037 AB Tilburg, The Netherlands; 3 University College London, Department of Security and Crime Science, London, WC1E 6BT UK

**Keywords:** Psychology, Society

## Abstract

Besides far-reaching public health consequences, the COVID-19 pandemic had a significant psychological impact on people around the world. To gain further insight into this matter, we introduce the Real World Worry Waves Dataset (RW3D). The dataset combines rich open-ended free-text responses with survey data on emotions, significant life events, and psychological stressors in a repeated-measures design in the UK over three years (2020: *n* = 2441, 2021: *n* = 1716 and 2022: *n* = 1152). This paper provides background information on the data collection procedure, the recorded variables, participants’ demographics, and higher-order psychological and text-derived variables that emerged from the data. The RW3D is a unique primary data resource that could inspire new research questions on the psychological impact of the pandemic, especially those that connect modalities (here: text data, psychological survey variables and demographics) over time.

## Background & Summary

Since the start of the pandemic, social and behavioural scientists have collected data on the psychological impact on individuals of COVID-19 and the measures introduced around it. The global health crisis severely impacted lives around the world. At the same time, it enabled social scientists across disciplines to study the response of humans to unprecedented circumstances. Several papers and associated datasets have emerged as a result of this, including those that adopted a psychological perspective. For instance, the *COVIDiSTRESS Global Survey* includes measures such as perceived stress, trust in authorities, and compliance with anti-COVID measures collected between 30 March and 30 May 2020 from 173,426 individuals across 39 countries and regions^1^. Similarly, the *PsyCorona* dataset consists of data collected at the start of the pandemic (*n* = 34,526) from 41 societies worldwide, measuring psychological variables and behaviours such as leaving the home and physical distancing^2^. That dataset has been used in follow-up studies to measure, for example, cooperation and trust across societies^3^ and associations between emotion and risk perception of COVID-19^4^. Others have studied the concept of ‘pandemic fatigue’ (i.e., the perceived inability to “keep up” with restrictions), for which there are data available from eight countries^5^. Associations between pandemic fatigue and the severity of restrictions were found, in addition to pandemic fatigue eliciting political discontent.

Of particular promise to understand how individuals fared during and in the aftermath of the pandemic are free-text responses, which allow for more depth and coverage of topics than targeted survey-style data collection. Some initiatives have used and made available linguistic data on the consequences of the pandemic, usually from Twitter^6,7^. In another study, Reddit and survey data were analysed to measure shifts in psychological states throughout the pandemic^8^. However, both modalities of data were collected from different participants, which does not allow for deeper exploration of ground truth psychological states of text authors by connecting survey and text modalities. Collecting text and survey data from the same participants is desirable for several reasons. Firstly, free-text responses enable participants to report their experiences in the pandemic in an unconstrained manner, potentially offering deeper insight into psychological processes. Secondly, simultaneously obtained survey responses offer ground truth measures on the psychological variables potentially underlying what is written about in text. Thirdly, advances made in the area of natural language processing allow for in-depth quantitative analyses of the text data, thereby making text data a resource that reaches beyond qualitative analyses typically conducted manually. However, collecting data that connect the textual dimension to survey data is costly as it requires primary data collection and cannot be realised through “found data” (e.g., posts on social media). Consequently, to date, such datasets are scarce and the lack thereof has impeded how we study the psychological impact of the pandemic.

The current paper fills that gap and introduces the *Real World Worries Waves Dataset (RW3D)* offering the unique combination of ground-truth survey data on emotions with free-text responses describing emotions in relation to the pandemic. The richness of this dataset allows us to examine, for example, emotional responses and the content of worries as a consequence of COVID-19. Given the broad scope of potential research questions and the scarcity and necessity of these data sources, we make this dataset available to the research community. Hereafter, we provide detailed background on the data collection procedure, recorded variables, participant demographics as well as an attrition analysis and descriptive statistics. We also provide evidence for latent clusters of how participants’ emotions changed over time and to what extent they were realistically or overly worried about various concerns in their lives. Our aim with this paper is to offer detail on a unique resource that could inspire plenty of research questions.

## Methods

### Ethics

The data collection was approved by the departmental ethics review board at University College London. No personal data were collected from participants and all participants provided informed consent for participation and for their data to be shared.

### Procedure

**Fig. 1:**
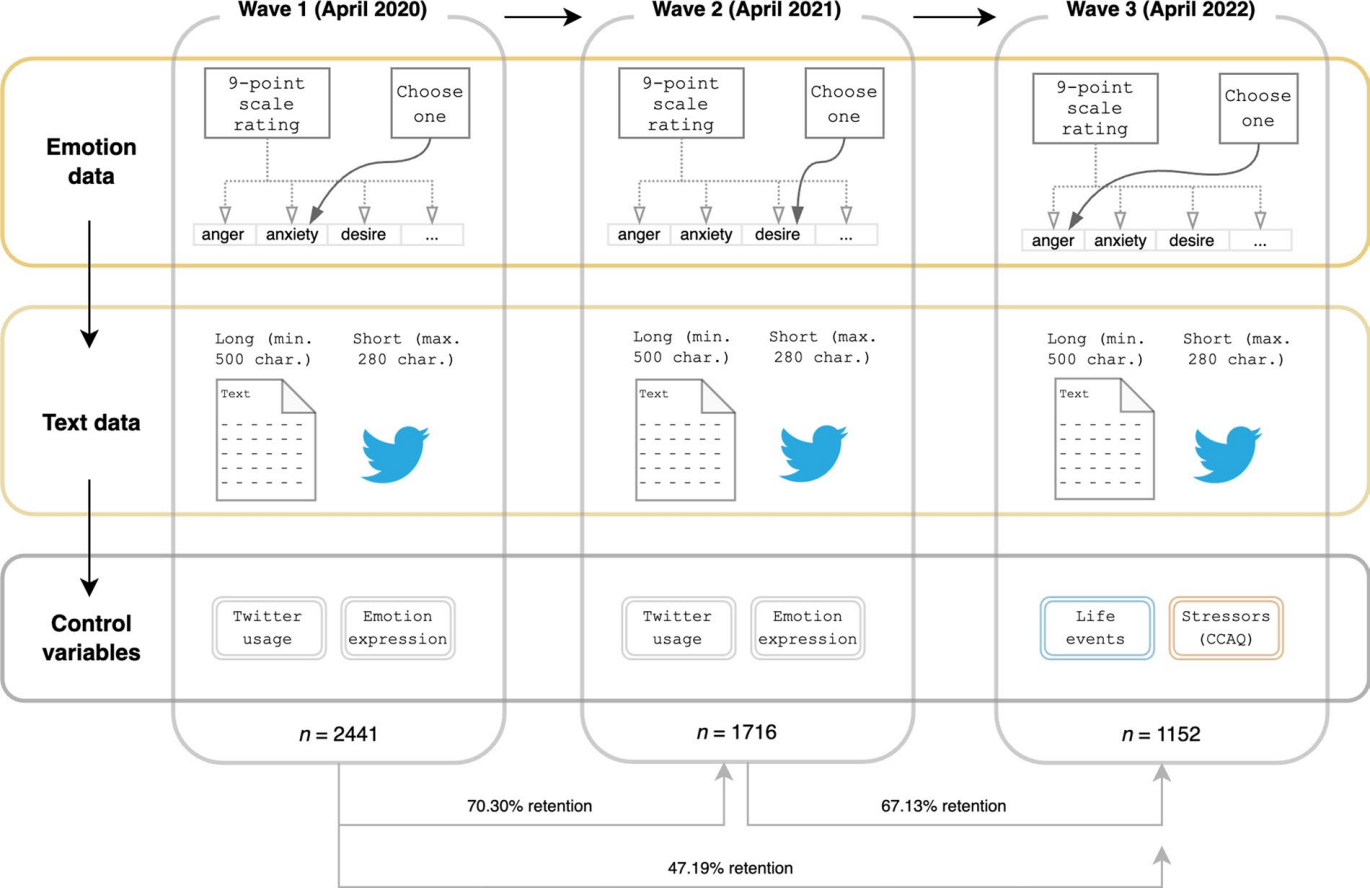
Data collection procedure and retention across waves.

The dataset was collected in three waves in April of 2020, 2021 and 2022. Data collection started in April 2020 on the crowdsourcing platform Prolific with an initial sample size of *n* = 2500. We then contacted the same participants through the crowdsourcing platform one year later about a follow-up data collection and made participation slots available for all participants whose data were collected in the first wave. That procedure was repeated another year later with those participants whose data were collected in wave 2. This resulted in sample sizes of *n* = 1839 in 2021, and *n* = 1227 in 2022. See Fig. 1 for an overview of the data collection procedure and retention across waves.

In all data collection phases, participants were informed about the purpose of the study, namely, to collect data about emotions and worries regarding the pandemic (see Supplemental Materials Table 1 for the full task intro and debrief). Participants started with the self-rated emotions questionnaire and the single emotion selection, then proceeded to the textual expressions and finally provided control variables (wave 1 and 2) and life events and psychological stressor variables (wave 3 only, see Fig. 1).

**Table 1:** Descriptive statistics per wave (M, SD) for the self-rated emotions (scale: 1 = not at all; 5 = moderately; 9 = very much) and the proportion of individuals who chose the respective emotion as “best fitting” emotion.

Emotion	*M*_Wave1_	*SD*_Wave1_	*prop.*_Wave1_	*M*_Wave2_	*SD*_Wave2_	*prop.*_Wave2_	*M*_Wave3_	*SD*_Wave3_	*prop.*_Wave3_
worry	6.67	1.70	—	5.07	2.03	—	3.98	2.16	—
anger	3.76	2.18	0.04	3.47	2.35	0.08	2.85	2.19	0.06
disgust	3.06	2.12	0.01	2.79	2.16	0.02	2.39	2.03	0.03
fear	5.63	2.30	0.09	3.77	2.30	0.02	2.85	2.05	0.02
anxiety	6.51	2.30	0.58	5.05	2.52	0.36	4.09	2.45	0.30
sadness	5.55	2.31	0.15	4.64	2.57	0.19	3.48	2.35	0.13
happiness	3.55	1.84	0.01	4.29	1.98	0.05	4.76	2.10	0.07
relaxation	3.83	2.05	0.12	4.54	2.25	0.23	5.14	2.35	0.38
desire	2.73	1.90	0.01	3.42	2.19	0.05	3.22	2.09	0.02

Only UK-based Prolific users who used Twitter (at least once a month) as per Prolific’s prescreening were eligible for participation. Upon completion of the survey, each participant was paid GBP 0.50. Even though the effective time spent on the task was somewhat longer than anticipated, we did not adjust the rewards so as not to introduce a change in reward as a confounding variable for the repeated-measures design. The task was administered through Qualtrics.

### Timing and societal context

The first wave of data collection occurred in early April 2020, when the UK was under lockdown with death tolls increasing. Queen Elizabeth II had just addressed the nation and then Prime Minister Boris Johnson was admitted to hospital due to COVID-19 symptoms^9^. In wave 2 (April 2021), many people in the UK had been vaccinated, and schools, retail and the hospitality sectors were (partially) re-opening. The delta-variant of the Coronavirus had just been identified at this time^10^. Finally, in wave 3 (April 2022) all travel restrictions for those entering the UK had been lifted, the Omicron variant was surging and news around the Partygate affair (i.e., a political scandal surrounding parties held at Downing Street during lockdown) was ongoing^11^.

### Demographic variables

We obtained participants’ demographics from Prolific. These are data that registered participants volunteered to provide and consist of their age, gender, country of birth, nationality, first language, employment status, student status, country of birth, country of residence as well as their participation on the crowdsourcing platform (number of tasks completed and approved). We have added one demographic question in the survey about their native language (as this may differ from their first language).

Participants were on average 37.10 years old (*SD* = 11.98) in April 2022, of which 68.4% were female (31.4% male, remaining: prefer not to say, see Supplemental Materials Table 2). The vast majority (90.5%) indicated the UK as their country of birth and as their current country of residence (99.7%), which matches the recruitment pre-selection that we made. Regarding their employment status, in 2020, 52.4% indicated being full-time employed, 22.7% in part-time work and 10.5% not in paid work (e.g., retired). Interestingly, the percentage of people in full-time work decreased somewhat in 2022 (42.4%). Similarly, the percentage of students decreased from 16.9% in 2020 to 10.9% in 2022.

### Emotion data

#### Self-rated emotions

Participants were asked to indicate on a 9-point scale how worried they were about the Corona situation (with labels at 1 = not worried at all; 5 = moderately worried; 9 = very worried) and how they felt at this moment about the Corona situation. For the latter, they indicated how strongly they felt each of the following eight emotions (1 = none at all; 5 = moderately; 9 = very much): anger, disgust, fear, anxiety, sadness, happiness, relaxation, desire^12^. The scale judgments were indicated using a slider in steps of 1 with labels at the extremes and in the middle for orientation.

#### Single emotion selection

Of the eight emotions listed above (i.e., excluding worry), each participant was asked “If you have to choose just one, which of the emotions below best characterises how you feel at this moment?”.

Table 1 shows the descriptive statistics for the emotion variables (self-rated scale values and discrete choice). While the pattern overall suggests improvement, in that the positive emotions increase while the negative ones decrease, there are latent patterns at play. Previous work using earlier waves of this data found clusters of participants in how their emotion scores changed from 2020 to 2021^10^ and we provide additional evidence for sub-groups below.

### Text data

We elicited two textual responses from each participant. The first text data were obtained through the following instruction: *“Please write in a few sentences how you feel about the Corona situation at this very moment. This text should express your feelings at this moment.”* Participants typed their response in a text field and received a prompt if their response was shorter than 500 characters. The second text response was obtained directly thereafter aimed at eliciting a shorter, Tweet-length text as follows: *“Suppose you had to express your current feeling about the Corona situation in a Tweet (max. 280 characters). Please write in the text box below”*. In this case, the participants were prompted if their text input was shorter than 10 or longer than 280 characters.

**Fig. 2:**
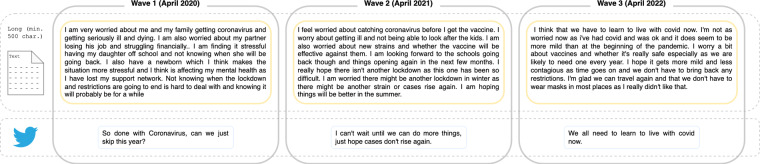
Text data of a single participant (long text and Tweet-size text).

The corpus descriptives (Supplementary Materials Table 3) show a stable length of both long and short texts over the three waves. In total, the corpus consists of 430,751 tokens (2020: 145,348; 2021: 144,191; 2022: 141,212). Figure 2 shows example texts written over all three waves.

### Control variables in wave 1 and 2

In the first two waves (April 2020 and 2021), we recorded two sets of control variables: the self-rated ability to express emotions in text and Twitter usage. We decided to drop these from the third wave. The rationale for dropping these variables was that we assumed these to change little within the individual and we already had two measurements (wave 1 and 2) that correlated substantially (see Table 2).

**Table 2:** Descriptives for the control variables collected in wave 1 and 2 (*M*, *SD*).

Variable	*M*_Wave1_	*SD*_Wave1_	*M*_Wave2_	*SD*_Wave2_	*r*
Emotion expression general	6.90	1.72	6.84	1.71	0.45 [0.40; 0.49]
Emotion expression short text	5.95	2.15	6.02	2.06	0.35 [0.30; 0.40]
Emotion expression long text	7.05	1.84	7.08	1.77	0.39 [0.34; 0.44]
Using Twitter	6.24	2.79	5.94	2.86	0.68 [0.65; 0.71]
Sending Tweets	3.77	2.55	3.45	2.49	0.73 [0.70; 0.75]
Conversations on Twitter	3.53	2.43	3.29	2.38	0.68 [0.65; 0.71]

All variables correlated substantially albeit somewhat stronger for the Twitter usage variables than for emotion expression ratings.

#### Emotion expression

As a potential control for the link between self-reported emotions in a survey and the expression of emotion in text, we asked participants to indicate on a 9-point scale (1 = not at all; 5 = moderately; 9 = very well) how well they (i) could express their feelings in general, (ii) how well in the Tweet-size text, and (iii) how well in the longer text.

#### Twitter usage

As an additional potential confounding variable specifically for the Tweet-size text we asked about participants’ Twitter usage. Using a 9-point scale (1 = never; 5 = every month; 9 = every day), participants indicated how often they (i) are on Twitter, (ii) send Tweets themselves, and (iii) participate in conversations on Twitter.

### New variables in wave 3

The most recent wave (April 2022) included two additional constructs that replaced the control variables from the previous waves. To better understand potential moderating variables of participants’ emotional adjustment in the pandemic and their textual expression, we collected data on important life events during the pandemic and used a crisis coping questionnaire^13^.

#### Life events

Participants were asked retrospectively about any important events or changes in their life that have happened to them over the past two years. First, they were asked whether *“anything - positive or negative - in [their] life [has] over the past two years impacted how [they] dealt with the Corona situation?”* Those who answered yes were then asked to describe the event, date the event (month and year) and rate the event’s impact on a scale from −10 (very negative) to +10 (very positive). If there was an additional event, participants could also submit one more (for a maximum of two events). All life events were subsequently qualitatively coded by the authors to arrive at overarching categories. For instance, being fired, changing jobs, and obtaining a first job after college were mapped to the category ‘job’; getting married, finding a partner, and a break-up were all mapped to ‘romantic’.

A third of the participants (33.9%) reported a significant life event during data collection. The most common life event category was ‘death’ (e.g., a death in the family), which was almost exclusively rated as a negative life event (97.6%). Life events related to work (e.g., a job change) were also common, which most participants (69.9%) rated with a positive intensity (Table 3). Other life events such as ‘mental health’ (e.g., experiencing panic attacks, receiving a mental health diagnosis) and ‘financial’ (e.g., paying off loans, loss of income) show a more ambivalent pattern and were rated as positive and negative with approximately equal proportion. Most life events occurred in December 2021 (median). See Table 4 for examples of each life event category.

**Table 3:** Summary of the life events data collected during the third wave with intensity (M, SD, Median) and proportion of participants who indicated a positive and negative intensity, between −10 (very negative) to +10 (very positive).

Event	Prop.	M (SD) intensity	Median intensity	Prop. neg. intensity	Prop. pos. intensity
no life event	66.15	NA	NA	NA	NA
death	7.20	−8.43 (2.83)	−10 [−10;6]	97.59	2.41
job	7.20	3.08 (6.3)	6 [−10;10]	28.92	69.88
romantic	3.12	−0.33 (7.95)	−4 [−10;10]	52.78	47.22
family	3.04	−1.88 (7.48)	−6 [−10;10]	65.71	37.14
reproduction	3.04	5.94 (5.97)	8 [−10;10]	17.14	82.86
health	2.52	−5 (6.38)	−8 [−10;10]	75.86	24.14
move	2.34	5.85 (4.93)	8 [−8;10]	11.11	88.89
health of family	1.91	−7.91 (2.58)	−8 [−10;0]	95.45	0.00
mental health	1.48	−1.18 (7.65)	−4 [−10;10]	52.94	47.06
education	0.69	−2.75 (5.23)	−5 [−8;4]	62.5	37.5
financial	0.61	−0.29 (7.61)	−4 [−10;10]	57.14	42.86
lifestyle	0.43	9.6 (0.89)	10 [8;10]	0.00	100.00
friendship	0.26	1.33 (9.87)	6 [−10;8]	33.33	66.67

**Table 4:** Examples (verbatim) of life events per category.

Event category	Example 1	Example 2	Example 3
death	We have lost 8 people, family and friends, to covid only one with underlying health condition. Very sad time.	Death of grandparents	My father passed away
job	My wife got a new job	I got a new job during lockdown which meant I was no longer forced into Work 5 days a week but stay at home half the week - I felt safer	Job loss due to government lockdown
romantic	Getting into a committed relationship	I have been dating the most beautiful woman ever.	breakup
family	Having to move back home and isolate caused me to realise how much I dont like my family	my son moved out	My Mother in Law accusing me of not letting her son have a vaccine.
reproduction	Grandughter born has given us reason to feel positive	2 years ago I had a baby	We had our 2nd child a few days before we went into the first national lockdown in March 2020
health	Getting Covid was a huge thing for me and it scared me enough to know that I never want to take risks and get it again having been in hospital with it.	Finally had an operation I had been waiting 2 1/2 years for.	I’ve gained a lot of weight and my health has suffered too quite a lot
move	I moved home to a new apartment,needed a change of scenery and life is good here.	I bought a house with my husband	moving to a different city
health of family	My father was ill, I live in Scotland and he lives in England. I have to be careful as I am immunocompromised, but seeing my father was more important than the risk to myself, so I travelled	My nan took seriously I’ll and we’ve missed 2 years of being able to visit and spend time with her she has dementia and now no longer remembers us	My father went blind and broke a bone in his back in a fall which robbed him of mobility as well as sight. It led to his latest fall and a positive COVID test.
mental health	I was diagnosed with depression.	I started taking antidepressants	I stopped caring and putting my time and energy into being invested in the daily news of corona. Made my anxiety and depression more stable.
education	Started teacher training Pgce	Going to university	Finishing uni
financial	Loss of income	Initial reaction of financial markets to the pandemic. I lost a large chunk of my retirement savings.	Work dried up for my partner and he is self employed so had an impact on our daily life.
lifestyle	I changed my lifestyle, started on a low-carb diet, started exercising and meditating and making time for myself.	It give me the push to do better with my running and lose some weight	The key thing is to be kind to myself. Try to eat healthily. Take some exercise where we can. I had stick to sleep routine. Pace myself. Take time to do the things I enjoy. Even if I can’t go outside.
friendship	The groups of friends I felt close by had moved away during the pandemic, in the country I had been living in for 3 years.	End of a relationship with a best friend, left one day and didnt see them again.	Break down of friendships

#### Stressors during crisis

To measure psychological stressors, we used a part of the Crisis Coping Assessment Questionnaire (CCAQ)^13^. Specifically, we asked several items from two perspectives: how *worried* they were about a range of concerns over the past two years (we refer to this below as the worry score) and how *problematic* each of the concerns turned out to be (the problem score). For each perspective, participants answered on a 9-point scale (1 = did not worry me at all/not problematic at all; 9 = worried me extremely/extremely problematic) to the following 12 concerns: their own physical health, mental health, and safety, the physical and mental health and safety of people they love, losing their job, not having enough money to survive, getting basic everyday things (food, etc.), social unrest, separation from their family, a close person being violent.

Responses to the CCAQ showed that participants were most worried about the physical safety and mental health of their loved ones. The extent to which these stressors occurred in reality showed that participants’ own mental health and that of their loved ones were impacted (Table 5). For all concerns measured, the worry score was never exceeded by the actual problem score. That is, participants were consistently more worried about an issue than that it turned out to be a problem. We see that such a worry-problem discrepancy is not evenly distributed across concerns from the CCAQ; below we provide evidence for two latent clusters of participants on that worry-problem discrepancy.

**Table 5:** Summary of worries about psychological stressors and how problematic each stressor turned out to be (M, SD) on a scale of 1–9 (1 = did not worry me at all/not problematic at all; 9 = worried me extremely/extremely problematic).

Variable	*M*_*worry*_	*SD*_*worry*_	*M*_*actual*_	*SD*_*actual*_
Own physical safety	5.16	2.23	3.58	2.34
Own mental state	5.69	2.40	4.92	2.63
Own safety	4.73	2.24	2.72	1.96
Physical safety loved ones	6.85	1.94	4.33	2.45
Mental health loved ones	6.31	2.09	4.77	2.40
Safety loved ones	6.22	2.20	3.57	2.36
Losing job	3.45	2.52	2.45	2.24
Financial problems	5.06	2.59	3.88	2.63
Getting basics	4.65	2.32	3.55	2.33
Social unrest	4.45	2.14	3.10	2.13
Being separated from family	5.13	2.63	4.13	2.65
Violence close person	1.67	1.49	1.44	1.30

## Data Records

The RW3D dataset is available on the Open Science Framework at https://osf.io/9b85r/^14^.The repository also contains all supplementary materials and a variable code book with detail and naming conventions for the full dataset.

The dataset contains columns for emotion ratings, long and short texts, linguistic metadata (number of characters, punctuation) and demographics separated per wave, indicated by the suffixes ‘_wave1’, ‘_wave2’, ‘_wave3’. For data collected in wave 3, we additionally provide - where applicable - up to two descriptions of life events and their associated impact ('life event’ variables), as well as all participants’ responses to the CCAQ scale (‘ccaq’ variables). Please see the codebook for a full description of each column.

## Technical Validation

This section describes (i) the steps taken to ensure data quality through participant exclusion criteria and (ii) how data derivatives were obtained.

### Data retention and exclusion

After each wave of data collection, we excluded participants based on two text-based criteria: if the long text was not written in the English language, as determined with the *cld* R package (https://cran.r-project.org/web/packages/cld3/index.html) or contained more than 20% punctuation tokens, participants were excluded. The latter was applied to remove participants who filled their textual response with superfluous continuous punctuation (e.g., dots, commas, exclamation marks) to reach the character length requirement. Both criteria were deemed necessary to ensure text data quality. For the third wave, the English-language criterion resulted in the exclusion of 38 participants and the punctuation criterion in a further four participants to be excluded (after the English-language criterion was already applied). The retention over the years was 70.3% and 67.1% in the second and third wave, respectively (see Supplementary Materials Table 2 for sample descriptives over the three waves).

### Data derivatives

We obtained two kinds of derivatives from the data, one based on the text data and the other on the emotion and CCAQ questionnaires. From the text data, we arrived at higher-order topics that provide an overarching theme for each written text and can be used to study what participants are writing about. The psychological variables (emotion scales and CCAQ) were mapped to higher-order psychological constructs characterised by latent clusters of participants on the emotion change (from 2020 to 2021 and from 2021 to 2022) as well as the discrepancy between their worry and problem score (i.e., the extent to which their worry about a concern was aligned with how problematic that concern turned out to be).

#### Topics

To capture overarching themes in the text data, we constructed a correlated topic model using the *stm* R package^15^ for the text data for each data collection wave. This probabilistic model is based on the assumption that a piece of text consists of a mix of topics, which in turn are a mix of words with probabilities of belonging to a topic^15,16^. Table 6 shows the top three most prevalent topics per wave for the long texts (see Supplementary Materials Tables 4, 5 for a full list of topics and terms for long and short texts). We have assigned labels to each topic based on the most frequent terms per topic.

**Table 6:** Top 3 most prevalent topics for the long texts in each wave, with assigned labels and most frequent terms.

wave	topic	% documents	terms
wave 1	rule following	10.29	peopl, feel, see, mani, die, govern, rule, think, will, follow
wave 1	how long will this last?	10.16	will, worri, feel, famili, normal, back, long, know, life, hope
wave 1	worry about loved ones	9.53	worri, also, famili, friend, time, anxious, work, home, feel, will
wave 2	hope for normality	15.04	will, feel, normal, hope, back, get, vaccin, thing, look, forward
wave 2	missing normality	14.38	work, want, friend, famili, feel, see, miss, time, abl, home
wave 2	anxiety	11.91	will, worri, vaccin, concern, also, feel, covid, anxious, effect, virus
wave 3	normal life	9.99	normal, back, now, life, live, covid, worri, feel, get, can
wave 3	still worried	8.70	covid, feel, still, worri, get, peopl, test, though, affect, don’t
wave 3	new variants	8.67	still, variant, vaccin, will, case, virus, new, concern, number, peopl

#### Higher-order psychological clusters

**Fig. 3:**
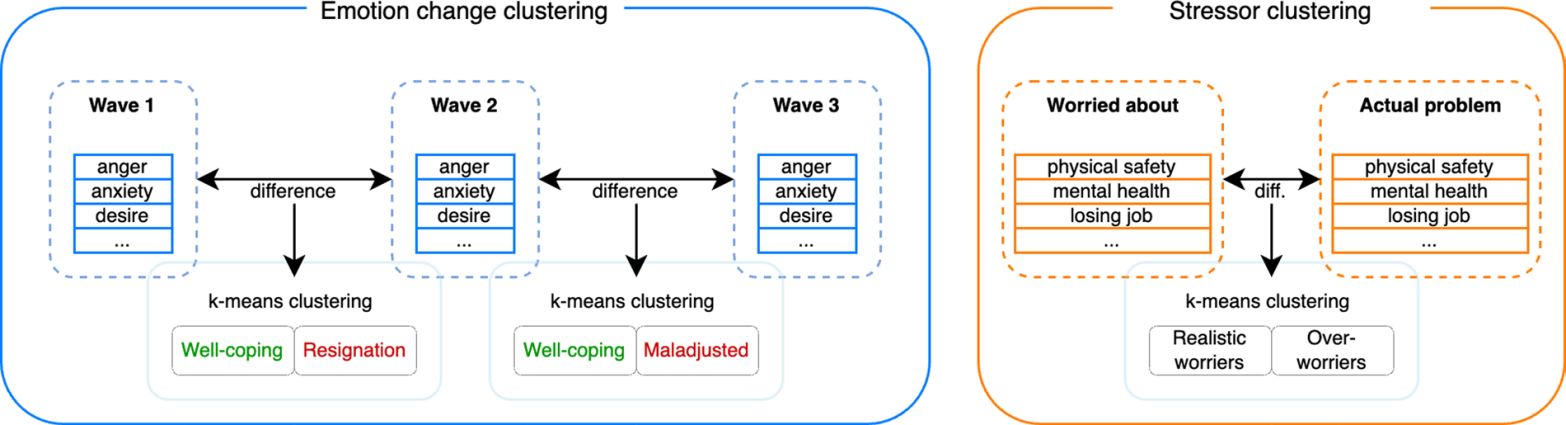
Clustering approach for two emotion changes and for the worry-problem discrepancy. The k-means algorithm showed an ideal number of clusters for *k* = 2 as per the Elbow method and the Silhoutte method.

Earlier work found evidence for latent clusters within the data in the change of emotions from wave 1 to wave 2^10^. We assessed whether there were additional emotion clusters in this extended dataset and also in the discrepancy between the worry score and the actual problem score on the concerns listed in the CCAQ. For each concept, we proceeded as follows (Fig. 3): we took the delta value of the emotion ratings for two time shifts (wave 2 minus wave 1, and wave 3 minus wave 2) and used the delta between the CCAQ worry score and problem score (worry minus actual problem score). For each change - emotion change from 2020 to 2021, emotion change from 2021 to 2022 - and the worry-problem discrepancy, we then ran k-means clustering^17^. We decided on the number of clusters through convergence of the scree plot and the Silhouette method. For all three delta values, there was evidence of two clusters.

The emotion clusters (Table 7) for the change from 2020–2021 were characterised by one group of participants (44.4% of the sample) showing a marked improvement in emotional well-being, while another group (55.6%) showed emotional responses resembling resignation (i.e., these participants reported higher anger, disgust and sadness but also lower worry and fear). The subsequent year’s clusters on the change from 2021 to 2022 showed again a well-coping group of participants (40.5%) with a very similar pattern to the earlier well-coping cluster, with the exception of no increase in desire. This was juxtaposed with a maladjusted group of participants (59.6%) who overlapped somewhat with the resignation cluster but which we termed differently due to this group’s increase in fear and anxiety and decrease in desire.

**Table 7:** Means per emotion for the latent emotion clusters at the 2020–2021 and 2021–2022 change (all sign. different from 0 at *p* < 0.01, two-sided, except for those with *ns*).

Emotion	2021 well-coping	2021 resignation	2022 well-coping	2022 maladjusted
anger	−1.68	0.83	−2.38	0.56
anxiety	−3.16	−0.11^*ns*^	−2.82	0.30
desire	1.24	0.25	0.27^*ns*^	−0.52
disgust	−1.26	0.53	−1.74	0.51
fear	−3.53	−0.53	−2.52	0.17
happiness	1.85	−0.14^*ns*^	1.65	−0.34
relaxation	2.21	−0.48	2.24	−0.53
sadness	−2.77	0.56	−3.27	0.28
worry	−2.40	−0.97	−1.79	−0.62
Size	44.4%	55.6%	40.5%	59.6%

A positive value denotes an increase in the respective emotion score in the later wave, while a negative value denotes a decrease. The two wellcoping clusters show similar patterns that suggest an increase in positive and a decrease in negative emotions. The resignation (2021) and mal-adjusted cluster show some overlap but differ in their change of desire and fear.

With regards to the worry-problem discrepancy clustering (Table 8), the larger of the two clusters (58.2%) was characterised by a markedly stronger “over-worry” (i.e., they indicated to worry about the various concerns much more than that they turned out to be a problem). Over-worrying was particularly evident on questions about the physical health and safety of loved ones. In contrast, the group that we termed the realistic worriers (41.8%) show consistently lower worry-problem discrepancies. Merely on the questions about someone close being violent (domestic violence) both groups were in agreement (low worry and low problem score).

**Table 8:** Clustering on the worry-problem discrepancy (all sign. different from 0 at *p* < 0.01, two-sided).

Variable	Realistic worriers	Over-worriers
Own physical safety	0.76	2.74
Own mental health	0.35	1.37
Own safety	1.17	3.17
Physical health loved ones	1.21	4.33
Mental health loved ones	0.69	2.72
Safety loved ones	1.35	4.47
Losing job	0.59	1.57
Financial problems	0.66	1.91
Getting basic needs	0.53	1.88
Social unrest	0.85	2.04
Separation from family	0.54	1.64
Domestic violence	0.22	0.24
Size	41.8%	58.2%

## Usage Notes

Understanding and addressing the psychological impact of the COVID-19 pandemic, and possibly preparing for the impact of future global crises, remains an ongoing research challenge. One of the impediments is high quality data that connects different modalities of how individuals experienced the pandemic. The current dataset paper introduced the RW3D, a repeated-measures dataset of UK participants, combining psychological variables examined via survey methods with rich textual responses. The explanatory relationships of coping in the pandemic are yet poorly understood. With the RW3D, we can examine via panel models to what extent life events, concerns raised in text data or socio-demographics changes (e.g., job loss) and variables (e.g., gender), explain changes over time into the various emotional response styles. Gaining insights into these complex relationships could also be a way forward to target interventions at those who most need it. Importantly, the inclusion of various control variables in the dataset allows researchers to control for potential confounds.

Moreover, we can also learn about some fundamental aspects of the relationship between text data and psychological variables. By connecting the modalities, we can test to what extent ground truth emotions are predictable from text data and whether a lagged design can help anticipate emotion changes at a later moment based on text data in previous years. Similarly, since we know about participants’ life events and stressors, we can assess how these are retrievable from the text data. One implicit assumption of plenty of applied text-based research is that these psychological variables are apparent from text data, but rich datasets to critically assess that assumption are scarce.

### Limitations

Some limitations need to be considered when making use of this dataset. First of all, the data were collected from UK participants only. While this allows for a rich analysis of the UK due to country-specific circumstances (e.g., infection spread, government responses), the results may not be generalisable to other populations. Second, some variables were collected retrospectively requiring participants to report significant life events up to 2 years after they happened and to think back about worries and actual problems of crisis coping. Third, while there is considerable spread in demographics, the dataset does not make use of nationally representative sample. A way to mitigate that concern post-hoc might be to weigh sample characteristics according to their prevalence in the UK population^18^.

### Supplementary information

Supplementary information

## Data Availability

There is no custom code associated with this data descriptor. For data(pre)processing and obtaining data derivatives, we used existing R packages. This included *cld* for English language checks, *quanteda*^19^ and *stringr* (https://stringr.tidyverse.org/) for text metadata (number of characters, tokens, punctuation), the *stm* package^15^ for constructing topic models, and the *factoextra* package (https://rpkgs.datanovia.com/factoextra/) for the determination of the number of clusters for obtaining the higher-order psychological constructs.
