# Supplementary material for: A multi-modal panel dataset to understand the psychological impact of the pandemic
Source: Sci Data. 2023 Aug 11;10:537. doi: 10.1038/s41597-023-02438-y (PMC10421916; doi:10.1038/s41597-023-02438-y)
Supplement: Supplementary file 1 — Supplementary information [file 41597_2023_2438_MOESM1_ESM.pdf]

Supplementary information for "The RW3D: A multi-modal panel dataset to understand the psychological impact of the pandemic"

Isabelle van der Vegt and Bennett Kleinberg

Table of contents

1. Task introduction and debrief
2. Basic sample characteristics
3. Corpus descriptives
4. Topics long texts
5. Topics short texts

**Intro**	"In this study, we ask you a few questions about how you feel about the corona (COVID-19) situation at this moment. We then ask you to write a brief text that expresses your current feelings. You are selected for this study because you may have participated in a similar study with us one a year ago. We are interested how your feelings have changed. This will help us understand how people are affected and about what. Your help and time is much appreciated. We hope our research can help us better understand how people are affected by the situation and how we can improve responses to it."
**Debrief**	"Thank you very much for your time and effort. In this study, we collected data about people's real worries about the current Corona situation in the UK. We hope that this will help us and other researchers to understand how we can help and inform better decision-making on a policy level."

Table 1. Task introduction and debrief.

**Wave**	**Initial**	**Age**	**Female (%)**	**Final**	**Retention (%)**
2020	2500	33.84	65.2	2441	100.00
2021	1839	36.22	67.4	1716	70.30
2022	1227	37.10	68.4	1152	67.13

Table 2. Basic sample characteristics per wave.

**Variable**	M_1	SD_1	$range_1$	M_2	SD_2	$range_2$	M_3	SD_3	$range_3$
Long: Tokens	126.17	39.37	[62, 1067]	125.17	32.05	[78, 387]	122.58	27.64	[62, 358]
Long: Chars.	624.20	196.66	[500, 5454]	621.27	158.19	[500, 2023]	610.82	133.98	[500, 1796]
Short: Tokens	26.63	15.90	[1, 75]	24.70	15.40	[1, 75]	24.59	14.62	[1, 67]
Short: Chars.	131.11	76.97	[10, 281]	121.64	75.12	[10, 281]	122.01	73.36	[10, 282]

Table 3. Corpus descriptives (M, SD, range) for all three waves of data collection. Note that the exceeding of 280 characters in the short texts is due to differences in counting leading and trailing white spaces

Table 4. Full list of topics for long texts per wave with topic prevalence (proportion). Please note the best fitting model for wave 1 has 15 topics (based on semantic coherence and exclusivity), whereas wave 2 and 3 have 10 topics.

wave	topic	proportion	terms
wave 1	Topic 2	10.29	peopl, feel, see, mani, die, govern, rule, think, will, follow
wave 1	Topic 10	10.16	will, worri, feel, famili, normal, back, long, know, life, hope
wave 1	Topic 7	9.53	worri, also, famili, friend, time, anxious, work, home, feel, will
wave 1	Topic 1	8.51	feel, time, get, home, work, can, situat, like, thing, news
wave 1	Topic 5	8.14	feel, worri, anxious, sad, get, day, like, famili, friend, see
wave 1	Topic 11	7.25	peopl, get, just, need, shop, realli, dont, much, home, day
wave 1	Topic 12	6.81	futur, worri, will, feel, anxious, situat, fear, also, get, health
wave 1	Topic 4	6.00	work, situat, worri, health, concern, feel, home, current, also, person
wave 1	Topic 8	5.94	peopl, take, serious, situat, feel, fear, hope, virus, health, get
wave 1	Topic 13	5.75	govern, nhs, angri, staff, feel, also, test, worker, peopl, social
wave 1	Topic 3	5.35	get, worri, will, shop, week, scare, abl, famili, food, virus
wave 1	Topic 9	5.17	feel, anxieti, children, anxious, famili, worri, can, know, see, one
wave 1	Topic 14	4.35	concern, virus, worri, death, corona, will, countri, lockdown, feel, get
wave 1	Topic 6	3.63	feel, due, peopl, health, sad, also, live, anxious, famili, home
wave 1	Topic 15	3.11	peopl, work, frustrat, famili, key, lockdown, arent, worker, rule, worri
wave 2	Topic 5	15.04	will, feel, normal, hope, back, get, vaccin, thing, look, forward
wave 2	Topic 7	14.38	work, want, friend, famili, feel, see, miss, time, abl, home
wave 2	Topic 3	11.91	will, worri, vaccin, concern, also, feel, covid, anxious, effect, virus
wave 2	Topic 8	11.49	feel, situat, life, like, anxious, will, pandem, futur, hope, normal
wave 2	Topic 9	11.08	peopl, virus, will, vaccin, still, rule, get, feel, think, mani
wave 2	Topic 4	9.32	feel, vaccin, still, hope, time, much, situat, lockdown, famili, now
wave 2	Topic 2	7.50	day, life, just, feel, get, dont, seem, want, know, lockdown
wave 2	Topic 1	7.40	govern, angri, peopl, anger, disgust, handl, vaccin, corrupt, death, nhs
wave 2	Topic 6	6.56	vaccin, get, feel, need, make, start, hope, will, still, school
wave 2	Topic 10	5.32	will, live, peopl, feel, lost, continu, get, just, one, safe
wave 3	Topic 1	14.29	get, back, normal, feel, now, thing, live, dont, just, life
wave 3	Topic 3	12.05	feel, peopl, still, covid, like, worri, situat, get, now, live
wave 3	Topic 6	11.79	still, vaccin, will, feel, variant, case, virus, concern, peopl, covid
wave 3	Topic 7	10.08	feel, peopl, mask, public, wear, govern, still, also, like, social
wave 3	Topic 9	9.41	will, feel, think, peopl, normal, time, back, rule, thing, work
wave 3	Topic 8	9.40	peopl, govern, test, covid, virus, will, vaccin, still, get, seem
wave 3	Topic 2	9.39	now, corona, think, virus, life, feel, live, peopl, normal, still
wave 3	Topic 4	9.35	worri, feel, know, covid, peopl, just, now, like, will, dont
wave 3	Topic 5	8.27	pandem, feel, now, live, covid, govern, time, situat, peopl, world
wave 3	Topic 10	5.98	covid, work, school, home, week, test, day, ill, children, last

Table 5. Full list of topics for short texts per wave with topic prevalence (proportion).

wave	topic	proportion	terms
wave 1	Topic 5	16.62	stay, home, peopl, everyon, safe, pleas, rule, other, insid, follow
wave 1	Topic 9	12.80	will, hope, life, just, now, normal, back, make, soon, fear
wave 1	Topic 10	10.51	situat, famili, sad, one, friend, worker, world, health, good, current
wave 1	Topic 8	10.18	feel, can, time, see, lockdown, posit, long, let, best, whole
wave 1	Topic 7	9.76	virus, want, peopl, think, countri, stop, frustrat, still, covid, angri
wave 1	Topic 3	8.78	get, thing, togeth, take, come, also, better, ever, scari, must
wave 1	Topic 4	8.39	anxious, live, nhs, work, help, save, dont, listen, much, wish
wave 1	Topic 2	8.04	worri, end, like, mani, tri, realli, affect, stress, covid-, pandem
wave 1	Topic 6	7.81	need, coronavirus, love, futur, day, risk, keep, put, lot, societi
wave 1	Topic 1	7.10	corona, scare, govern, know, look, miss, happen, possibl, job, month
wave 2	Topic 10	12.34	hope, can, famili, keep, let, safe, way, nhs, dont, even
wave 2	Topic 9	12.27	normal, back, peopl, look, forward, anxious, sad, school, right, frustrat
wave 2	Topic 4	11.68	vaccin, now, see, life, time, virus, fed, home, bore, happi
wave 2	Topic 6	11.05	end, lockdown, corona, light, come, tunnel, long, final, summer, well
wave 2	Topic 8	10.81	covid, govern, need, everyon, day, work, start, pleas, protect, everyth
wave 2	Topic 7	9.81	get, still, want, rule, soon, one, follow, optimist, mani, lost
wave 2	Topic 5	9.52	thing, wait, situat, cant, friend, return, abl, take, make, never
wave 2	Topic 3	8.54	feel, will, futur, coronavirus, worri, world, stop, ever, sick, wear
wave 2	Topic 2	7.09	live, pandem, stay, much, think, yet, chang, children, good, near
wave 2	Topic 1	6.90	just, year, like, better, realli, mask, tire, posit, done, number
wave 3	Topic 7	13.68	peopl, mask, govern, wear, think, seem, other, mani, public, health
wave 3	Topic 9	13.08	feel, life, will, can, pandem, situat, hope, one, new, start
wave 3	Topic 8	11.99	covid, now, year, vaccin, dont, worri, look, know, forward, two
wave 3	Topic 10	11.96	still, normal, thing, keep, happi, safe, pleas, take, everyon, want
wave 3	Topic 2	8.76	need, restrict, protect, case, vulner, work, good, lockdown, anymor, abl
wave 3	Topic 1	8.63	corona, virus, care, way, long, make, yet, must, ever, next
wave 3	Topic 5	8.62	live, back, just, see, away, rise, world, current, bit, covid-
wave 3	Topic 3	7.87	coronavirus, continu, glad, move, learn, never, final, fear, last, rememb
wave 3	Topic 4	7.84	like, test, much, around, isol, realli, free, better, posit, longer
wave 3	Topic 6	7.59	get, time, stay, let, gone, famili, isnt, enjoy, serious, done
